# Supplementary material for: Patterns of Intron Gain and Loss in Fungi
Source: PLoS Biol. 2004 Nov 30;2(12):e422. doi: 10.1371/journal.pbio.0020422 (PMC532390; doi:10.1371/journal.pbio.0020422)
Supplement: Table S1 — Also available at http://genes.mit.edu/NielsenEtAl/. (4.3 MB ZIP). [file pbio.0020422.st001.zip › NielsenEtAl/html/1214.html]

AN6870.1.NCU04672.1.MG10534.1.FG02207.1


```
 CLUSTAL W (1.82) Multiple Sequence Alignments - Introns Inserted


Sequence 1: NCU04672.1	531 aa
Sequence 2: MG10534.1	501 aa
Sequence 3: FG02207.1	496 aa
Sequence 4: AN6870.1	507 aa
Alignment Length: 535 aa
Number Identitical Residues: 245 aa
Alignment Score (without introns) 11933


MG10534.1 	MPPSISS---RSATAHEEEAIETTALLPR----------QQQQQQHRSRAEYAAWLREIW
NCU04672.1	MSGATPS---RSADLLKPRPDETASLVRKPPSLQS-SSTQVEESEVPNLPEYKLWLSELW
FG02207.1 	MTSQHSPSIEPSYRILESFPDEETPLIPH----------SRNSSLEIKSSSRSPWLTETV
AN6870.1  	MYSSIHTT-APGPEYDSPTPTEYTSLLPKPTDDLFPDSLSSRDAEVTDHDSRKAILAEFL
          	*     .:   .    .  . * :.*: :...   ..: . ..    .  .    * *  

MG10534.1 	FLTKACVPVILAYMLQNSLQTISVLIVGRLSPEALSAAAFAYMFAMATAWLIALGGTSAL
NCU04672.1	LLTKNAIPVIFAYTLQNSLQTGSILIVGRLSPEALATAAFSYMFAMSTAWLIALGGSTAL
FG02207.1 	VLVKASVPVILAYMLQNSLQTVSVLMVGRLSPEALATAAFSYMFTMATAWLIALGGTTAL
AN6870.1  	LLLKSSVPVILAYALQNSLQTVSVLIVGRISPEHLATSAFSLMFAMITAWMIALGGTTAL
          	.* * .:***:** ******* *:*:***:*** *:::**: **:* ***:*****::**

MG10534.1 	DTLASSSFTRSRDKTSLGLLLQRGFLVLTALYAVVVVVWWFAEDVFRALGQPEEICVMSA
NCU04672.1	DTLASSSYTGSSNKHDLGILLQRGLAVLSALYAVMALIWTFSEPLFRVLGQEEYICVQSA
FG02207.1 	DTLGSSTFTSSKDKHDLGILLQRGLIVLTGFYGVVAIIWCFSGYIFRSLGQEEFICVQGP
AN6870.1  	DTLASSSFTGS-DKRNLGILLQRGFFVLGLFYLPVAVLWIFSEKVFLLLGQDADLSKDSA
          	***.**::* * :* .**:*****: **  :*  :.::* *:  :*  ***   :.  ..

MG10534.1 	RFLRLLAPGGLGYVWFECMKKFLQAQA~IYRPGTYALMLTSPLNALLNYLLIYKLDMGLD
NCU04672.1	MFLRRLIPGGLGYIWFECMKKFLQAQE1VYRPGTYVLLITSPLNALLNYFLIHKLGLGLH
FG02207.1 	RFLQLLIPGGLGYVWFEAMKKFLQAQE~IYRPGTYALLVISPLNAVLNWFFIYTLDFGLD
AN6870.1  	QFLTALIPGGLGYVYFELMKKYLQAQG1IMRPGTYVLLLTSPLNAALTYTFCYKLGLDLL
          	 **  * ******::** ***:****  : *****.*:: ***** *.: : :.*.:.* 

MG10534.1 	GAPIATGISYWLSFFFLVGYAVFVQGSECWGGFDLRRAATNVGPFARLAALGVVHVGTEW
NCU04672.1	GAPLATGISYWCSFFLLVAYATFVRGHECWGGLSPHRALSNIGPFIRLALLGIIHVGTEW
FG02207.1 	GAPIATGISYWLCFLLLVGYTAFIKGSACWGGFNIRQALQHLGPFSKLAFLGVIHVGTEW
AN6870.1  	GAPLALGISYWLSFALLVLYAILVRGSECWGGWS-REALTNLGTFSRLAILGVVHVGTEW
          	***:* ***** .* :** *: :::*  **** . :.*  ::*.* :** **::******

MG10534.1 	WAFEIVALAAGRLGTLPLAAQSVIMTADQIINTIPFGLGVAESARLGNLLGAGDVRGARR
NCU04672.1	WAFEIVALAAGQLGTIALAAQSVIMTADQIINTIPFGLGVAASSRVGNLLGARKVKEASR
FG02207.1 	WAFEIVALAAGRLGTIPLAAQSVVMTLDQIINTIPFGLGVAASSRLGNLLGLGDAVQARR
AN6870.1  	WAFEIVALAAGRLGTIPLAAQSVIMTADQVLNTIPFGVGVATSARVGNLLGAKDAKGARR
          	***********:***:.******:** **::******:*** *:*:*****  ..  * *

MG10534.1 	SAHSAAILSVVFGTTLLVVLMAARHRVGALFNDDEQVIALVAEVVPYVALFQIADGLNGS
NCU04672.1	ASHCAVVLSIVAGALILTVLMSVKDVFGRLFNDDERVIRLVADVMPFVALFQIADGLNGS
FG02207.1 	CAHSAALLSIIFGTIILTVLLSTKNVIAKIFNDDEEVVTLVAHIMPYVALFQIADGLNGS
AN6870.1  	AANVAAWLSMILGLVVLVAFMGTKDHFAKIFNSDPRVVSLTSAVLPFVALFQIADGLNGS
          	.:: *. **:: *  :*..::..:. .. :**.* .*: *.: ::*:*************

MG10534.1 	CGGALRGMGRQWVGALVNCVSYYGGALPGGIYLAFNGWGLAGLWMGQCVALSLVGVLEWV
NCU04672.1	CGGVLRGTGRQWVGAAVNLVSYYGGALPTGIYLAFHGWGLRGLWIGQCVALYLVGVLEWL
FG02207.1 	CGGALRGMGRQWVGAVVNCISYYGGALPAGIWLAFHGWGLSGLWVGQCVALFLVGALEWV
AN6870.1  	CGGSLRGMGRQHVGAAVNIVSYYCGALPLGVYLAFHGWGLKGLWVGQCVALYLVGALEWV
          	*** *** *** *** ** :*** **** *::***:**** ***:****** ***.***:

MG10534.1 	IVGMSDWPEEVRRAIDRLRDDEDGN------------KSVVEEEVVE---------
NCU04672.1	IVGMSNWEDEVKRAISRLDDGGFGDSSAHVYAYGAIGEGQADEALGERHREGREPV
FG02207.1 	IIALCDWKKEVKRASDRIR------------------VDDLDRNL-----------
AN6870.1  	IVAASDFDKEVRKAFKRMDEAAY--------------AEEEEGRL-----------
          	*:. .:: .**::* .*: .                     :  :
```
